# Preoperative Prediction of Inferior Vena Cava Wall Invasion of Tumor Thrombus in Renal Cell Carcinoma: Radiomics Models Based on Magnetic Resonance Imaging

**DOI:** 10.3389/fonc.2022.863534

**Published:** 2022-06-06

**Authors:** Zhaonan Sun, Yingpu Cui, Chunru Xu, Yanfei Yu, Chao Han, Xiang Liu, Zhiyong Lin, Xiangpeng Wang, Changxin Li, Xiaodong Zhang, Xiaoying Wang

**Affiliations:** ^1^ Department of Radiology, Peking University First Hospital, Peking University, Beijing, China; ^2^ Department of Nuclear Medicine, Sun Yat-Sen University Cancer Center, Guangzhou, China; ^3^ State Key Laboratory of Oncology in South China, Collaborative Innovation Center for Cancer Medicine, Guangzhou, China; ^4^ Department of Urology, Peking University First Hospital, Institute of Urology, Peking University, National Urological Cancer Center, Beijing, China; ^5^ Department of Radiology, The Second Affiliated Hospital, Zhejiang University School of Medicine, Hangzhou, China; ^6^ Beijing Smart Tree Medical Technology Co. Ltd, Research and Development Department, Beijing, China

**Keywords:** carcinoma, renal cell, thrombus, vena cava, inferior, magnetic resonance imaging, radiomics

## Abstract

**Objective:**

To develop radiomics models to predict inferior vena cava (IVC) wall invasion by tumor thrombus (TT) in patients with renal cell carcinoma (RCC).

**Methods:**

Preoperative MR images were retrospectively collected from 91 patients with RCC who underwent radical nephrectomy (RN) and thrombectomy. The images were randomly allocated into a training (n = 64) and validation (n = 27) cohort. The inter-and intra-rater agreements were organized to compare masks delineated by two radiologists. The masks of TT and IVC were manually annotated on axial fat-suppression T2-weighted images (fsT2WI) by one radiologist. The following models were trained to predict the probability of IVC wall invasion: two radiomics models using radiomics features extracted from the two masks (model 1, radiomics model_IVC; model 2, radiomics model_TT), two combined models using radiomics features and radiological features (model 3, combined model_IVC; model 4, combined model_TT), and one radiological model (model 5) using radiological features. Receiver operating characteristic (ROC) curve analysis and decision curve analysis (DCA) were applied to validate the discriminatory effect and clinical benefit of the models.

**Results:**

Model 1 to model 5 yielded area under the curves (AUCs) of 0.881, 0.857, 0.883, 0.889, and 0.769, respectively, in the validation cohort. No significant differences were found between these models (*p* = 0.108-0.951). The dicision curve analysis (DCA) showed that the model 3 had a higher overall net benefit than the model 1, model 2, model 4, and model 5.

**Conclusions:**

The combined model_IVC (model 3) based on axial fsT2WI exhibited excellent predictive performance in predicting IVC wall invasion status.

## Introduction

Renal cell carcinoma (RCC) is the most common renal malignancy, accounting for nearly 4% of all malignancies (1). Some patients with RCC are initially diagnosed with advanced tumors since these patients rarely exhibit the typical triad of hematuria, side pain, and abdominal tumor. Stage T3 RCC with venous extension can form a tumor thrombus (TT) (2), with approximately 10% reaching the level of the inferior vena cava (3); 1% of the thrombus may even grow into the right atrium (4). The Mayo staging system divides the cephalic extent of the TT into five levels based on optimal surgical planning (4). For patients with RCC with TT in the IVC, if regional lymph node metastasis and distant metastasis are not detected preoperatively, radical nephrectomy and thrombectomy remain the only chance for survival (5–7). European Urology Association (EAU) (3) current guidelines recommend that surgical resection of non-metastatic RCC with IVC thrombosis be considered in patients with acceptable performance status. This recommendation is also endorsed by the National comprehensive cancer Network (NCCN) guidelines (1). However, there is a higher cancer-specific mortality rate among patients with TT invading the IVC wall (8). If the IVC wall is not invaded, the TT can be milked back through the venous lumen. If the IVC wall is invaded, segmental resection and IVC reconstruction are generally necessary (4, 9, 10). The choice of surgery is typically determined by intraoperative exploration. Some authors have pioneered techniques for laparoscopic and robotic management of RCC with IVC thrombosis. Several practices (11, 12) have demonstrated that minimally invasive surgery is technically feasible to achieve acceptable perioperative outcomes. In clinical practice, it is very important to accurately judge whether the IVC is invaded by TT before operation, thus benefiting for the evaluation operation difficulty evaluation and the operation plan formulation.

Imaging examination is an essential tool for preoperative prediction of the status of the IVC wall, providing a lot of morphological information to help clinicians better prepare for surgery. Some studies have confirmed that magnetic resonance imaging (MRI) is the best preoperative examination for evaluating the extension of TT and the IVC wall invasion among all imaging modality (13). Several signatures based on MRI have been proposed for predicting invasion of the IVC wall with good histopathological correlation (14–16). Among them, a large volume of the TT and subjective features of complete occlusion of the IVC lumen, irregular margin of the TT, thickened IVC wall, and abnormal signal of the IVC wall are the most common (14, 15, 17–19). However, subjective evaluation is empirically dependent and has achieved only a moderate degree of consistency and accuracy. Furthermore, the various proposed manual measurement methods are cumbersome. Alayed et al. (15) proved that texture analysis technique can be used to judge the intrusion of IVC wall, but the data amount is small. More recently, radiomics, with its use of high-throughput-derived imaging features, has demonstrated the potential to be a convenient method for personalized oncology management (20). We hypothesized that radiomics could help to determine the presence or absence of invasion of the IVC wall pathologically. Thus, the aim of the present study was to develop radiomics models to predict inferior IVC wall invasion by TT in patients with RCC.

## Materials and Methods

This retrospective single-center study was reviewed and approved by our hospital [IRB number: 2019 (170)] with a waiver of informed consent.

### Study Sample

The images and medical records of patients who underwent radical nephrectomy and thrombectomy for IVC due to RCC in our hospital were retrospectively collected from January 2010 to December 2020. The inclusion criteria were: a) the patients underwent MRI examination to evaluate the TT and underwent radical nephrectomy and thrombectomy within one month after an MR examination. b) Complete sequences and qualified images. The exclusion criteria were: a) neoadjuvant chemotherapy, radiotherapy, or interventional therapy for RCC before MR examination or surgery; b) the coronal T2-weighted images (T2WI) or gadolinium-enhanced T1-weighted images (T1WI) include the upper or lower boundaries of the whole TT. c) incomplete surgical or pathological records; d) renal mass was pathologically confirmed as non-RCC.

**Figure 1:**
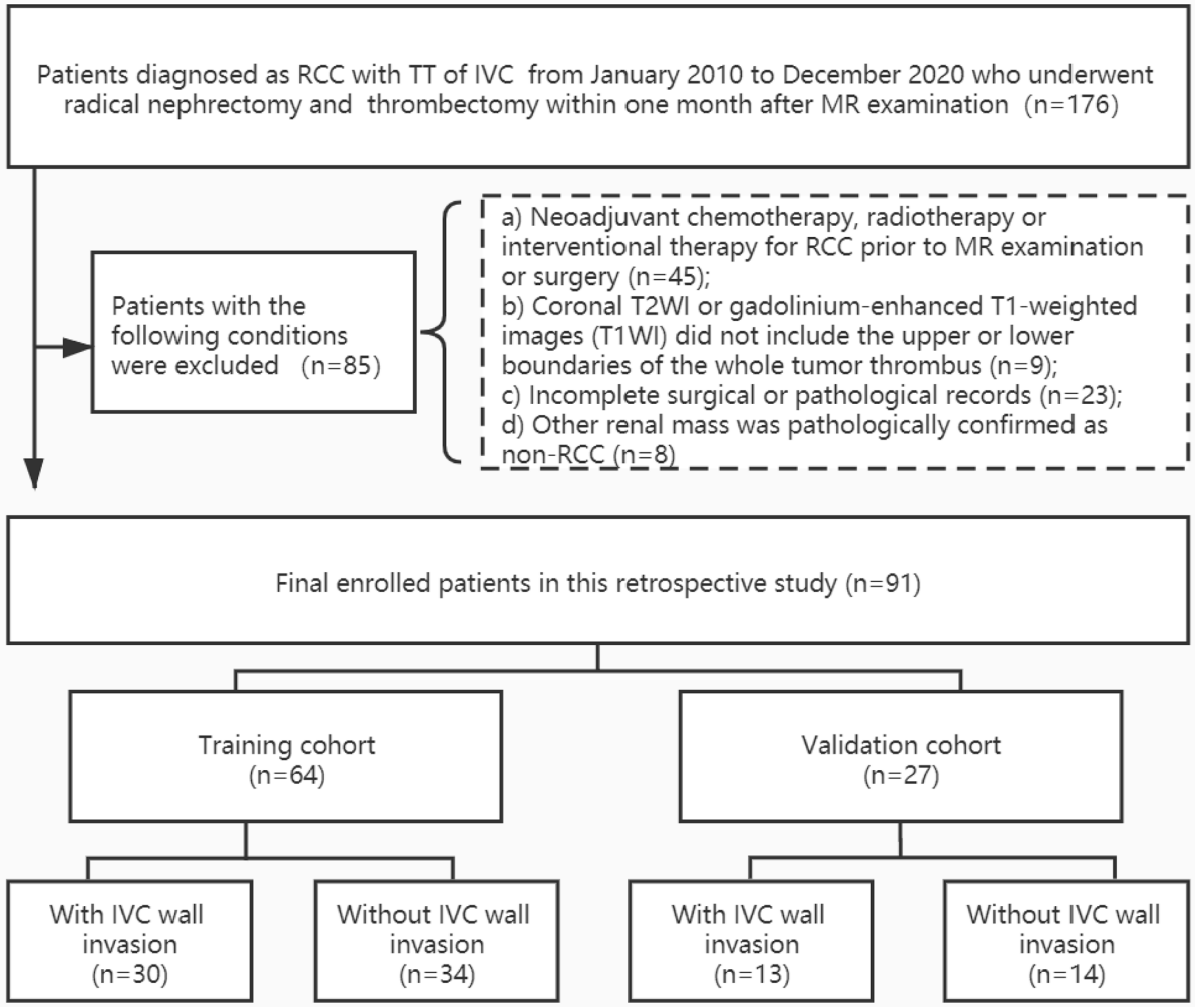
Patient enrollment folw chart. RCC, renal cell carcinoma; TT, tumor thrombus; IVC, inferior vena cava. Other renal mass include four angiomyolipoma, one inflammatory myofibroblastic tumor, one synovial sarcoma, one malignant solitary fibrous tumors, and one primitive neuroectodermal tumor.

In total, 91 eligible patients (69 men and 22 women; median age, 56[49,64] years) were included. Patients were randomly assigned into a training cohort (n = 64) and a validation cohort (n = 27) at a ratio of 7:3. The training cohort was used to construct the various models and the validation cohort was used to test the predictive performance of each model. Demographic information, as well as operative and histopathologic records, were archived from medical records. **Figure 1** illustrates the patient enrolment process.

### MR Imaging Acquisition

Patients underwent abdomen MR examinations using either a 3.0-Tesla scanner (Scanner 1: Discovery HD 750, Ge Healthcare, Milwaukee, WI) or a 1.5-Tesla scanner (Scanner 2: Signa Twinspeed; GE, USA) with phased-array coils. The standard protocol at our institution included: a) fat-suppression T2WI in the axial and coronal planes; b) axial in-phase and out-of-phase T1WI; c) axial diffusion-weighted imaging (DWI) with the reconstruction of apparent diffusion coefficient (ADC) maps; and (d) dynamic contrast-enhanced (DCE) MRI if gadolinium contrast is injected.

In this study, only axial fat-suppression T2WI was used for radiomics analysis. All the sequences were used for radiological analysis. The detailed acquisition parameters for the fat-suppression T2WI are presented in **Supplementary Material S1** and **Table S1**.

### Reference Standards for Invasion

The invasive samples and non-invasive samples were histologically verified and intraoperatively confirmed according to the electronic medical records system. IVC wall invasion was pathologically confirmed by segmental or circumferential resection of the TT. If the TT was too large to resect, the surgeon’s intraoperative observation was considered as evidence of TT invasion of the IVC. If no adhesion was found during operation and the TT was easily removed, it is considered non-invasive.

### Radiological Analysis

Two experienced radiologists (Z.S. and X.L., both with more than three years of expertise in genitourinary MR interpretation) reviewed the images together to assess the following features: quantitative measurements of TT (craniocaudal extent, maximal anterior-posterior diameter of RV, maximal superior-inferior diameter of RV, maximal anterior-posterior diameter of IVC, and maximal coronal diameter of IVC) and the presence or absence of several subjective features (irregular margin of TT, thickening of IVC wall, occlusion of the IVC, abnormal signal intensity on T2WI). Both radiologists were blinded to the clinical details of the patients. In case of disagreement, a third senior radiologist (X.W., with more than 20 years of experience) confirmed the findings. A radiological model was constructed using the final confirmed radiological features.

### Region of Interest Masking

**Figure 2:**
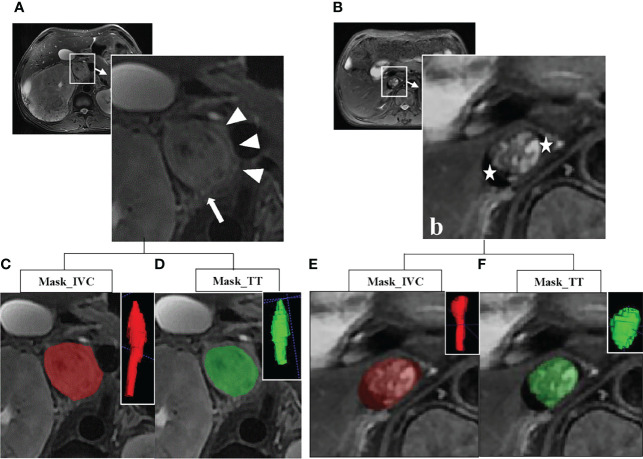
**(A)** Axial fat-suppression T2-weighted image (fsT2WI) in a 68-year old man with a clear cell renal cell carcinoma (cRCC) and an inferior vena cava (IVC) tumor thrombus (TT) with wall invasion. Subjective features of complete occlusion of the IVC lumen, the irregular margin of the TT (arrow), thickened IVC wall (arrow and triangle), and abnormal signal of the IVC wall (arrow) can be found. **(B)** Axial fsT2WI in a 76-year old woman with cRCC and a TT in IVC without wall invasion. The crescent-shaped black areas on the laterodorsal aspect of IVC stand for flow void (star). **(C–F)** Examples for annotation of IVC and TT. The red represents the mask of the IVC, and the green represents the mask of the TT. The three-dimensional (3D) volumes of interest (VOIs) are at the upper right corner.

The ITK-SNAP Toolbox v 3.6.0 (http://www.itksnap.org) was used to annotate the IVC and TT on axial fsT2WI section by section. As shown in **Figure 2**, the volumes of interest (VOIs) included the outer margin of the TT or IVC and avoided extra-lesion structures. In the craniocaudal extent, the entire length of the IVC, including where there is no TT, was annotated.

The agreement of inter-reader and intra-reader was preliminarily evaluated with 45 randomly chosen samples for VOIs-based radiomics features extraction by the two radiologists (reader 1 Z.S. and reader 2 X.L.) in a blinded fashion. To assess intra-reader reproducibility, reader 1 performed the VOIs delineation twice with an interval of more than four weeks. Meanwhile, reader 2 performed the VOIs delineation only once to evaluate the inter-reader reproducibility by comparing with the first results of reader 1. The Dice similarity coefficient (DSC) was used to determine the spatial overlap accuracy of VOIs (21). The DSC ranged from 0 to 1, with a high value indicating better agreement. The reproducibility of radiomics features was evaluated with the interclass correlation coefficient (ICC). An ICC of greater than 0.75 was considered to represent good reproducibility. The workflow for the remaining samples was completed by Reader 1. In the end, the mean slices annotated for IVC and TT were 23.03 ± 3.49 and 9.98 ± 4.22.

### Radiomics Analysis and Modeling

The modeling workflow included the following steps: a) feature extraction, b) model construction, and c) predictive performance validation. The radiomics features were extracted using the PyRadiomics package in Python (22). A total of 1070 radiomics features were extracted from each mask, including 216 first-order statistical features, 14 shape-based features, and 840 texture features (**Supplementary Material S2** and **Table S2**). Combined models were constructed using radiomics features and radiological features. In this study, four types of normalization methods, two types of dimension reduction methods, four types of feature selectors, and ten types of classifiers were used for modeling (**Supplementary Materials S3, S4,** and **Table S3**). To sum up, a total of 6,400 (4 × 2 × 4 × 20 × 10) models were established for each mask through the permutations and combinations of each step. The validation cohort was used to evaluate the predictive effect of the four models for IVC wall invasion. All processes of construction and validation of the radiomics models were implemented in Python (v 3.6.0).

### Statistical Analysis

The Mann-Whitney U test, independent samples t-test, and chi-square test were used for univariate analyses under the appropriate circumstances. Significant predictors from the univariate analyses were included in a binary logistic regression analysis to explore the variables with statistically significant differences in predicting IVC wall invasion. Receiver operating characteristic (ROC) curve analysis was conducted to calculate the area under the ROC curve (AUC), sensitivity, specificity, and accuracy of each model. Multiple and pairwise comparisons of AUCs were achieved using the DeLong nonparametric approach (23). A decision curve analysis (DCA) was used to analyze the net benefits of the different models. Statistical analysis was performed with R 3.5.1 (Comprehensive R Archive Network, www.r-project.org), SPSS 24.0 (SPSS Inc., Chicago, IL, USA), and MedCalc 15.8 (MedCalc Software, Ostend, Belgium). A two-sided *p* <.05 was considered statistically significant.

## Results

### Patient Characteristics

The demographic and clinical characteristics of the patients are summarized in **Table 1**. There were no significant differences between the training cohort and the validation cohort in terms of age, sex, side, histopathologic diagnoses, Mayo classification, Fuhrman grade, rhabdomyolysis, or sarcomatoid degeneration, objective measurements, and subjective characteristics (*p* = 0.058-0.924). In 4 cases, the TT was observed to invade the IVC wall, and the operation was abandoned due to the complexity. A total of 57 patients underwent segmental or circumferential resection of the TT, among which 40 cases were pathologically confirmed to be infiltrated by tumor cells, and 17 cases were not infiltrated by tumor cells. No adhesion to the IVC wall was found in the remaining 30 cases during operation, which was easy and complete to remove. Thus, 48% (44/91) of the cases were assigned to the IVC wall invasion group and 51.6% (47/91) were assigned to the IVC wall non-invasion group (**Figure 1**).

**Table 1:** Demographic and clinical characteristics in the training cohort and validation cohort.

	Training cohort (n=64)			Validation cohort (n=27)			*P* ^†^
	Invasion (n=31)	No invasion (n=33)	*P*	Invasion (n=13)	No invasion (n=14)	*P*	
Age, mean ± SD (years)	52.5 ± 11.3	55.1 ± 10.7	0.754	52.1 ± 13.7	58.2 ± 9.0	0.130	0.686
Male, n (%)	29 (93.5)	20 (60.6)	0.002	20	12	0.587	0.255
Mean kg/m^2^ body mass index (range)	24.8 (21.1-28.8)	25.1 (17.5-28.7)	0.734	24.0 (21.0-25.8)	24.5 (21.0-28.7)	0.807	0.026
Right kidney involvement, n (%)	17 54.8)	26 (78.8)	0.043	10 (76.9)	10 (71.4)	0.749	0.924
Histopathologic diagnoses, n (%) Clear cell carcinoma Papillary carcinoma Chromophobe cell carcinoma Collecting duct carcinoma Others	26 (83.9) 5 (16.1) 0 (0.0) 0 (0.0) 0 (0.0)	25 (75.8) 2 (6.1) 2 (6.1) 2 (6.1) 2 (6.1)	0.280	11 (84.6) 2 (15.4) 0 (0.0) 0 (0.0) 0 (0.0)	13 (92.9) 0 (0.0) 0 (0.0) 0 (0.0) 1 (7.1)	0.563	0.352
Mayo classification^#^, n (%) I II III IV	0 (0.0) 14 (45.2) 11 (35.5) 6 (19.4)	7 (21.2) 20 (60.6) 3 (9.1) 3 (9.1)	0.001	3 (23.1) 4 (30.8) 3 (23.1) 3 (23.1)	4 (28.6) 8 (57.1) 2 (14.3) 0 (0.0)	0.149	0.058
Fuhrman Grade, n (%) 1 2 3 4	1 (3.2) 5 (16.1) 19 (61.3) 6 (19.4)	0 (0.0) 11 (33.3) 18 (54.5) 4 (12.1)	0.214	0 (0.0) 0 (0.0) 10 (76.9) 3 (23.1)	0 (0.0) 4 (28.6) 7 (50.0) 3 (21.4)	0.224	0.838
Rhabdomyolysis or sarcomatoid degeneration, n (%)	7 (22.6)	4 (12.1)	0.271	3 (23.1)	2 (14.3)	0.564	0.295
3.0-Tesla scanner, n(%)	15 (48.4)	23 (69.7)	0.214	7(53.8)	7(50.0)	0.867	0.510
Craniocaudal extent, mean ± SD (cm)	7.59 ± 3.47	5.10 [2.80,7.65]	0.024*	9.47 ± 4.23	3.61 ± 2.88	<0.001	0.599
Maximal anterior-posterior diameter of RV, mean ± SD (cm)	1.78 ± 0.57	1.712 ± 0.52	0.633	1.80 [1.60, 2.10]	1.63 ± 0.68	0.189^‡^	0.686
Maximal superior-inferior diameter of RV, mean ± SD (cm)	1.85 ± 0.49	1.867 ± 0.62	0.915	1.90 [1.75, 2.70]	1.79 ± 0.75	0.253	0.484
Maximal anterior-posterior diameter of IVC, mean ± SD (cm)	3.13 ± 1.041	2.358 ± 1.04	0.004*	3.31 ± 0.93	1.60 [1.12, 3.23]	0.006	0.758
Maximal coronal diameter of IVC, mean ± SD (cm)	3.797 ± 0.97	2.555 ± 1.05	<0.001*	3.37 ± 0.89	2.29 ± 1.10	0.01	0.201
Irregular margin of tumor thrombus, n (%)	21 (67.7)	4 (12.1)	<0.001*	13 (100.0)	2 (14.3)	<0.001	0.150
Thickening of IVC wall, n (%)	17 (54.8)	7 (21.2)	<0.001*	8 (61.5)	1 (7.1)	0.003	0.707
Occlusion of the IVC wall, n (%)	24 (77.4)	9 (27.3)	<0.001*	11 (84.6)	2 (76.9)	<0.001	0.767
Abnormal signal intensity on T2WI, n (%)	23 (74.2)	1 (3.0)	<0.001*	12 (92.3)	3 (21.4)	<0.001	0.538

Data in parentheses are percentages and data in brackets are interquartile range (IQR).

SD, standard deviation; IVC, inferior vena cava; RV, renal venous.

^†^Comparison between the training cohort and the validation cohort.

*Predictors included in the Binary logistic regression model (p <.05).

^#^The level of tumor thrombus was classified as 0 (thrombus limited to the renal vein, detected clinically or during the assessment of the pathological specimen), I (thrombus extending<2 cm above the renal vein), II (thrombus extending >2 cm above the renal vein, but below the hepatic veins), III (thrombus at the level of or above the hepatic veins but below the diaphragm), and IV (thrombus extending above the diaphragm).

### Construction of the Radiological Model

The results of the univariate analyses are shown in **Table 1**. In the training cohort, the craniocaudal extent, the maximal anterior-posterior diameter of the IVC, maximal coronal diameter of the IVC, irregular margin of TT, thickening of the IVC wall, occlusion of the IVC, and abnormal signal intensity on T2WI were associated with IVC wall invasion (p<0.05) and were used as predictors in a binary logistic regression model to predict IVC wall invasion by TT. Independent predictors in binary logistic regression are detailed in **Table 2**. Equations to calculate the probability of IVC wall invasion were generated at **Supplementary Material S5**.

**Table 2:** Independent predictors with the radiological model for inferior vena cava wall invasion in the training cohort.

Parameters	*β*	Odds ratio (95%CI)	*p*
Irregular margin of tumor thrombus	1.736	5.673 (1.104, 29.144)	0.038*
Abnormal signal intensity on T2WI	3.949	51.887 (5.751, 468.142)	0.000*
Constant	-1.812	0.163	0.000*

Data in parentheses are 95% confidence interval.

β indicates the regression coefficient; CI, confidence interval.

*Represents statistically significant.

### Inter-Reader and Intra-Reader Agreements of Vois and Radiomic Features

Inter-reader agreement of VOIs achieved a mean DSC value of 0.909 (95% CI, 0.900–0.920) and 0.911 (95% CI, 0.887– 0.917) for VOI_IVC and VOI_TT. Intra-reader agreement of VOIs achieved a mean DSC value of 0.975 (95% CI, 0.967–0.975) and 0.956 (95% CI, 0.933–0.960) for VOI_IVC and VOI_TT. Inter-reader reproducibility of radiomic features reached a mean ICC value of 0.961 (95% CI, 0.957–0.966) and 0.977 (95% CI, 0.973–0.981) for VOI_IVC and VOI_TT. Intra-reader reproducibility of radiomic features reached a mean ICC value of 0.993 (95% CI, 0.989–0.995) and 0.977 (95% CI, 0.973–0.981) for VOI_IVC and VOI_TT.

Construction of radiomics models and combined models

The models with the highest AUC values in the training cohort were taken as the results for the model 1 (radiomics model_TT), model 2 (radiomics model_IVC), model 3 (combined model_TT), and model 4 (combined model_IVC), respectively (**Supplementary Material Table S4**). The normalization, dimension reduction, feature selectors, and classifiers of each model are detailed in **Table 3** and **Supplementary Materials S3, S4**.

**Table 3:** Modeling pipelines of the radiomics models and combined models.

	Radiomics model_IVC	Radiomics model_TT	Combined model_IVC	Combined model_TT
Normalization	None	Z-score	None	Mean
Dimension reduction	PCA	PCC	PCA	PCC
Feature selection	KW	ANOVA	KW	ANOVA
Classification	XGB	DT	DT	RF

PCA, principal component analysis; PCC, Pearson correlation coefficient; ANOVA, analysis of variance; KW, Kruskal–Wallis test; XGB, eXtreme. Gradient Boosting; DT, Decision Tree.

### Validation of Radiological Model, Radiomics Models, and Combined Models

**Figure 3:**
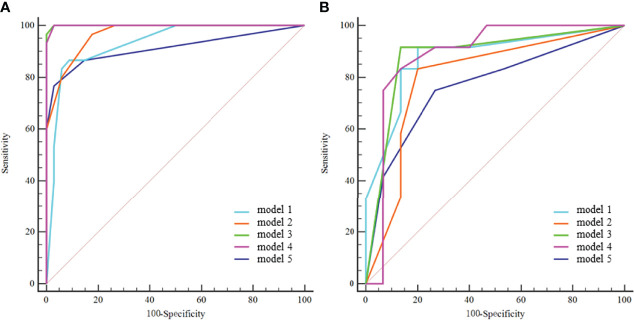
ROC curves of the five models in the training cohort **(A)** and validation cohort **(B)**. model 1 = radiomics model_IVC; model 2= radiomics model_TT; model 3 = combined model_IVC; model 4 = combined model_TT; model 5 = radiological model.

Model 1 to model 4 yielded AUCs of 0.931, 0.967, 1.000, and 0.999, respectively, in the training cohort and 0.881, 0.857, 0.883, and 0.889, respectively, in the validation cohort for the prediction of IVC wall invasion (**Figure 3**). The model 5 (radiological model) yielded an AUC of 0.912 in the training cohort and 0.769 in the validation cohort. The AUC, accuracy, sensitivity, and specificity of all five models in the validation cohort are shown in **Table 4**.

**Table 4:** The five models’ performance in predicting inferior vena cava wall invasion by tumor thrombus.

	AUC (95% CI)	SEN (95% CI)	SPE (95% CI)	ACC(95% CI)	Threshold
**Training cohort** model 1 model 2 model 3 model 4 model 5	0.931 (0.839, 0.979) 0.967 (0.889, 0.996) 1.000 (0.943, 1.000) 0.999 (0.942, 1.000) 0.912 (0.815, 0.968)	0.867 (0.693, 0.962) 0.967 (0.828, 0.999) 1.000 (0.884, 1.000) 1.000 (0.884, 1.000) 0.767 (0.577, 0.901)	0.912 (0.763, 0.981) 82.35 (0.655, 0.932) 0.971 (0.847, 0.999) 0.971 (0.847, 0.999) 0.971 (0.847, 0.999)	0.875 (0.770, 0.938) 0.890 (0.788, 0.949) 0.984 (0.909, 1.000) 0.984 (0.909, 1.000) 0.860 (0.752, 0.927)	0.484 0.250 0.556 0.300 0.850
**Validation cohort** model 1 model 2 model 3 model 4 model 5	0.881 (0.698, 0.973) 0.857 (0.669, 0.961) 0.883 (0.701, 0.974) 0.889 (0.708, 0.976) 0.769 (0.568, 0.908)	0.917 (0.615, 0.998) 0.846 (0.516, 0.979) 0.917 (0.615, 0.998) 0.833 (0.516, 0.979) 0.750 (0.428, 0.945)	0.800 (0.519, 0.957) 0.857 (0.519, 0.957) 0.867 (0.595, 0.983) 0.867 (0.595, 0.983) 0.733 (0.449, 0.922)	0.741 (0.551, 0.871) 0.741 (0.551, 0.871) 0.889 (0.711,0.970) 0.852 (0.669, 0.947) 0.630 (0.442, 0.785)	0.370 0.556 0.556 0.400 0.481

The data shown in brackets represent the 95% confidence intervals (CIs).

model 1 = Radiomics model_IVC; model 2= Radiomics model_TT; model 3 = Combined model_IVC; model 4 = Combined model_TT; model 5 = Radiological model; AUC, area under the curve; ACC, accuracy; SEN, sensitivity; SPE, specificity.

**Figure 4:**
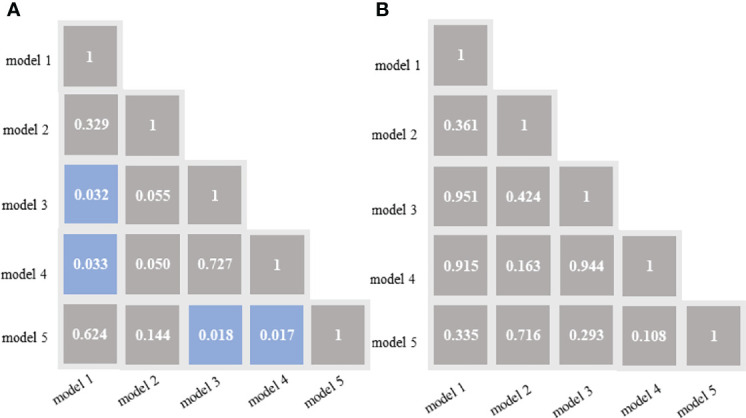
DeLong test of the areas under the curve (AUCs) of the 5 models in the training cohort **(A)** and validation cohort **(B)**. Blue boxes represent p<0.05; Other grey boxes show p > 0.05. model 1 = radiomics model_IVC; model 2= radiomics model_TT; model 3 = combined model_IVC; model 4 = combined model_TT; model 5 = radiological model.

In the training cohort, the DeLong test showed that both the model 3 and model 4 outperformed the model 5, and the differences were statistically significant. Both the model 3 and model 4 outperformed the model 1, and the differences were statistically significant (**Figure 4A**). In the validation cohort, the AUCs of the five models were pairwise compared by the DeLong test. The differences were not statistically significant (*p*=0.108-0.951) (**Figure 4B**).

**Figure 5:**
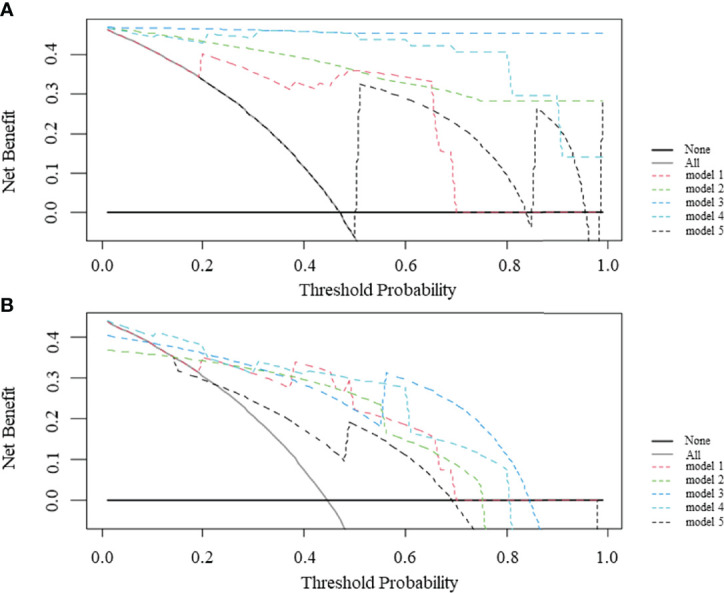
Decision curve analysis (DCA) comparing the net benefits of different models in the training cohort **(A)** and validation cohort **(B)**. The y-axis measures the net benefit and the x-axis indicates the threshold probability. model 1 = radiomics model_IVC; model 2= radiomics model_TT; model 3 = combined model_IVC; model 4 = combined model_TT; model 5 = radiological model.

**Figure 5** illustrates the dicision curve analysis (DCA) of the overall utility of the model 1 to model 5 in the training cohort and validation cohort. In the validation cohort, all models acquired higher net benefits compared to the all-treat or none-treat protocol. The model 1 to model 4 showed higher net benefits than the model 5 with different threshold probabilities. The DCA showed that the model 3 had a higher overall net benefit than the model 1, model 2, model 4, and model 5. The two combined models (model 3 and model 4) had higher net benefits than the radiomics models (model 1and model 2), based on either the IVC or TT as the mask.

## Discussion

Preoperative evaluation of the status of IVC wall invasion by TT in patients with RCC is crucial for treatment planning and prognosis. In this study, based on fsT2WI, two radiomics models and two combined models were developed to predict IVC wall invasion. The models were compared with the radiological model.

The radiological model included variables reflecting previously described imaging signs and quantitative measurements among 91 cases. Although MR is regarded as the optimal examination method, it is still a challenge for radiologists to evaluate IVC invasion. Using the imaging signs and quantitative measurements on MRI, radiologists’ evaluations of IVC wall invasion have a sensitivity and specificity of 63.6-100.0% and 86.4-92.3%, respectively (14, 15, 17). In this study, the radiological model incorporating irregular margin of TT and abnormal signal intensity on T2WI also yielded a moderate sensitivity of 75.0% and specificity of 73.3%. The combined model_IVC exhibited the best prediction performance with a sensitivity of 91.7% and specificity of 86.7%, which was better than the radiological models. Our study differed from those of previous studies and have strength in three aspects. First, we are the first to apply recently emerging radiomics analysis techniques to the prediction of IVC wall invasion. Secondly, we have a significant advantage in sample size, and the prediction result of the radiological model is better than those previously reported. Thirdly, we compare the effectiveness of several radiomics models and radiological model, and conclude that the radiomics models are superior to the radiological model.

Radiomics techniques can extract numerous quantitative features from a mask which can reveal gray-level patterns, spectral patterns, and pixel interrelationships; this means that the selection of the mask is critical. This paper sheded some light at an innovative topic of radiomics. Different mask may play a diffferent role in the model performance In this study, the regions of the IVC and TT were respectively selected as masks to construct two radiomics models. Our preliminary results of the comparison of the radiomics models based on the different mask showed that the radiomics model based on mask_IVC outperformed the radiomics model based on mask_TT. Xie et al. (24) developed three radiomics models based on different masks to differentiate uterine sarcoma from leiomyoma and found that the prediction performance of the three models was different. Adding clinical or radiologist interpretation information may improve the radiomics model. In the current study, we tried to add radiologists’ interpretations to radiomics features extracted from the two masks to build two combined models. There were no significant differences in the two combined models incorporating radiological interpretations compared to the radiomics models alone. Previous studies have demonstrated that adding clinical information or radiological features can improve the effectiveness of radiomics (25–28). Although there was no statistically significant difference in the AUCs of the models, the DCA showed a similar conclusion, suggesting the possible superiority of the combined model. However, this should be confirmed in the future in studies with larger sample sizes.

Axial fsT2WI were chosen to develop the models in the current study, for three reasons. First, these images can clearly show the TT and IVC and can be used to easily identify their boundaries. Second, axial fsT2WI is a routine sequence available in every RCC patient evaluated by preoperative MR, and thus, the sample size was somewhat adequate. Third, the features extracted from fsT2WI may be repeatable. Lecler et al. studied the repeatability of multiple sequences of lacrimal gland MR images and extracted a total of 145 radiomics features. The results showed that more repeatable radiomics features were found in fsT2WI and enhanced T2WI images (44% and 31%) (29) than other sequences including axial T1-WI, axial Diffusion-WI, coronal DIXON-T2-WI, and coronal post-contrast DIXON-T1-WI.

At present, few studies have applied the radiomics technique to explore the invasion of the IVC wall by TT. Alayed et al. attempted to use the texture analysis technique to analyze MR images of 24 patients with RCC complicated with TT of the IVC. They found larger entropy in the group with IVC wall invasion but there were no significant differences in the other texture parameters (15). In constructing the radiomics model_TT and combined model_TT, several shape features and textural features were selected, which suggests that the invasiveness of TT is related to the size and heterogeneity of the TT. The selection of the features was similar to some of the quantitative measurement indices and subjective features proposed by previous studies (14, 15, 17–19).

In the validation cohort, the DCA curve was significantly better than that of the radiological model. Therefore, from the perspective of clinical application, the effectiveness of the radiomics models is better than the experienced radiologist. However, the AUC value of the radiomics models in this study ranged from 0.881 to 0.889, which indicates that it would not be reliable enough to avoid surgery. In real clinical practice, urologists should not only refer to the results of this model but also integrate more comprehensive clinical information.

The current study has several limitations. The retrospective design and single-center data may introduce some bias. In the future, by increasing the amount of data and optimizing the algorithm to improve the stability of the model, prospective experiments can be considered for inclusion in the study. The current models apply to axial fsT2WI; other images can be explored in the future. Manually annotated IVC and TT were adopted in this study, which is time-consuming. In the future, we can try to select an image that best reflects the invasion of the IVC for analysis, rather than annotation section by section. Or we might explore the feasibility of automatic segmentation by deep learning method to obtain the region of interest to avoid manual annotation. The axial fsT2WI were obtained from two MR scanners (3.0T and 1.5T systems) at random, which may be considered a limitation of the radiomics techniques. There are minor changes to the MR scan protocol (**Supplementary Table S1**) during the study started in 2010 and ended in 2020. It is worth mentioning that, from a medical point of view, there is no morphological difference in tumors between 3.0T and 1.5T systems. The subtle differences of images caused by different magnetic field intensities and different scanning protocols may also improve the generalization ability of radiomics models. This is supported by prior studies (24, 26, 30) that have attempted to construct radiomics models with images from 3.0-Tesla and 1.5-Tesla systems. Due to the low incidence of RCC with TT, the total number of cases included is limited to fit the principle of events per variable (EPV) (31). The sample size in our study is the maximum we could obtain practically. To ensure generalizability, more data will need to be included for model construction in the next step.

## Conclusion

In summary, the promising results of the current study show the possibility of predicting IVC wall invasion status by radiomics, and the combined model_IVC based on axial fsT2WI exhibited good predictive performance.

## Data Availability Statement

The raw data supporting the conclusions of this article will be made available by the authors, without undue reservation.

## Ethics Statement

The study involving human participants were reviewed and approved by Biomedical Research Ethics Committee of Peking University First Hospital [IRB number: 2019 (170)]. The ethics committee waived the requirement of written informed consent for participation.

## Author Contributions

XiaoW and ZS conceived of the presented idea. CX, YY, and CH collected the original data. XL, ZS, and ZL analyzed the data. ZS drafted the manuscript. YC and XZ finished the picture production. XiangW and CL provided algorithm support. All authors reviewed the manuscript and XiaoW made corrections to the manuscript. All authors contributed to the article and approved the submitted version. All authors contributed to the article and approved the submitted version.

## Conflict of Interest

The reviewer JL declared a shared parent affiliation with the author(s) CX, YY, XL, ZL, XZ and XW to the handling editor at the time of review.

Author XW and CL were employed by Beijing Smart Tree Medical Technology Co. Ltd.

The remaining authors declare that the research was conducted in the absence of any commercial or financial relationships that could be construed as a potential conflict of interest.

## Publisher’s Note

All claims expressed in this article are solely those of the authors and do not necessarily represent those of their affiliated organizations, or those of the publisher, the editors and the reviewers. Any product that may be evaluated in this article, or claim that may be made by its manufacturer, is not guaranteed or endorsed by the publisher.
