# Supplementary material for: Preoperative Prediction of Inferior Vena Cava Wall Invasion of Tumor Thrombus in Renal Cell Carcinoma: Radiomics Models Based on Magnetic Resonance Imaging
Source: Front Oncol. 2022 Jun 6;12:863534. doi: 10.3389/fonc.2022.863534 (PMC9207178; doi:10.3389/fonc.2022.863534)
Supplement: Supplementary file 1 [file DataSheet_1.docx]

**S1: Acquisition parameters**

The detailed acquisition parameters for the fat-suppression T2WI are presented in Table S1.

**Table S1.** Magnetic resonance sequence parameters

|  | 3.0-Tesla scanner | 1.5-Tesla scanner |
| --- | --- | --- |
| Plane | Axial | Axial |
| TR/TE | 11000~12000 / 80~100 | 6000~8000 / 80~100 |
| Band width, KHz | 83.3 | 31.2 |
| Section thickness, mm | 5~6 | 5~6 |
| Intersection gap, mm | 1~1.5 | 1~1.5 |
| FOV, cm^2^ | 40 | 40 |
| Matrix | 512 ×512 | 512 ×512 |

TR= repetition time; TE= echo time; FOV= field of view.

**S2: Radiomics feature extraction**

To extract the radiomics features, we performed the following 3 steps related to the parameter setting (1):

1. Three settings are specified as follows: “binWidth”, “label”, and “voxelArrayShift”.

2. Three image types are enabled (“Original”, “LoG” (Laplacian of Gaussian) and “Wavelet”), with custom settings specified for “LoG” (“sigma: [1.0, 3.0, 5.0]”) and “Wavelet” (“[LLH, LHL, HLL, LHH, HHL, HLH, HHH, LLL]”).

3. The following six feature classes are defined: “shape”, “firstorder”, “glcm”, “glrlm”, “glszm” and “gldm”; the classes are enabled with all possible features in the respective class.

The following three groups of radiomics features were used (2): first-order statistical features (n=18), shape-based features (n=14), and textural features (n=24 GLCM +16 GLRLM +16 GLSZM +14 GLDM) (Table S2).

Therefore, 216 ((1 Original + 3 LoG + 8 Wavelet) ×18) first-order statistical features, 14 shape-based features and 840 ((1 Original + 3 LoG + 8 Wavelet) × (24+16+16+14)) texture features (for a total of 1070 (216+14+840) radiomics features) were extracted from each volume of interest (VOI).

**Table S2 Radiomic features used in this study**

|  |  | Features | |
| --- | --- | --- | --- |
| **First-order Statistical Features** |  | 10^th^ Percentile | |
| **(n=18)** |  | 90^th^ Percentile | |
|  |  | Energy | |
|  |  | Entropy | |
|  |  | Interquartile Range | |
|  |  | Kurtosis | |
|  |  | Maximum | |
|  |  | Mean Absolute Deviation | |
|  |  | Mean | |
|  |  | Median | |
|  |  | Minimum | |
|  |  | Range | |
|  |  | Robust Mean Absolute Deviation | |
|  |  | Root Mean Squared | |
|  |  | Skewness | |
|  |  | Total Energy | |
|  |  | Uniformity | |
|  |  | Variance | |
| **Shape Features** |  | Elongation |  |
| **(n=14)** |  | Flatness | |
|  |  | Least Axis Length | |
|  |  | Major Axis Length | |
|  |  | Maximum 2D Diameter (Column) | |
|  |  | Maximum 2D Diameter (Row) | |
|  |  | Maximum 2D Diameter (Slice) | |
|  |  | Maximum 3D Diameter | |
|  |  | Mesh Volume | |
|  |  | Minor Axis Length | |
|  |  | Sphericity | |
|  |  | Surface Area | |
|  |  | Surface Volume Ratio | |
|  |  | Voxel Volume | |
| **Textural Features: Gray Level Co-occurrence Matrix (GLCM) Features** |  | Autocorrelation | |
| **(n=24)** |  | Cluster Prominence | |
|  |  | Cluster Shade | |
|  |  | Cluster Tendency | |
|  |  | Contrast | |
|  |  | Correlation | |
|  |  | Difference Average | |
|  |  | Difference Entropy | |
|  |  | Difference Variance | |
|  |  | Inverse Difference (ID) | |
|  |  | Inverse Difference Moment (IDM) | |
|  |  | Inverse Difference Moment Normalized (IDMN) | |
|  |  | Inverse Difference Normalized (IDN) | |
|  |  | Informational Measure of Correlation (IMC) 1 | |
|  |  | Informational Measure of Correlation (IMC) 2 | |
|  |  | Inverse Variance | |
|  |  | Joint Average | |
|  |  | Joint Energy | |
|  |  | Joint Entropy | |
|  |  | Maximal Correlation Coefficient (MCC) | |
|  |  | Maximum Probability | |
|  |  | Sum Average | |
|  |  | Sum Entropy | |
|  |  | Sum of Squares | |
| **Textural Features: Gray Level Run Length Matrix (****GLRLM) Features** |  | Gray Level Non-Uniformity (GLN) | |
| **(n=16)** |  | Gray Level Non-Uniformity Normalized (GLNN) | |
|  |  | Gray Level Variance (GLV) | |
|  |  | High Gray Level Run Emphasis (HGLRE) | |
|  |  | Long Run Emphasis (LRE) | |
|  |  | Long Run High Gray Level Emphasis (LRHGLE) | |
|  |  | Long Run Low Gray Level Emphasis (LRLGLE) | |
|  |  | Low Gray Level Run Emphasis (LGLRE) | |
|  |  | Run Entropy (RE) | |
|  |  | Run Length Non-Uniformity (RLN) | |
|  |  | Run Length Non-Uniformity Normalized (RLNN) | |
|  |  | Run Percentage (RP) | |
|  |  | Run Variance (RV) | |
|  |  | Short Run Emphasis (SRE) | |
|  |  | Short Run High Gray Level Emphasis (SRHGLE) | |
|  |  | Short Run Low Gray Level Emphasis (SRLGLE) | |
| **Textural Features: Gray Level Size Zone Matrix (****GLSZM) Features** |  | Gray Level Non-Uniformity (GLN) | |
| **(n=16)** |  | Gray Level Non-Uniformity Normalized (GLNN) | |
|  |  | Gray Level Variance (GLV) | |
|  |  | High Gray Level Zone Emphasis (HGLZE) | |
|  |  | Large Area Emphasis (LAE) | |
|  |  | Large Area High Gray Level Emphasis (LAHGLE) | |
|  |  | Large Area Low Gray Level Emphasis (LALGLE) | |
|  |  | Low Gray Level Zone Emphasis (LGLZE) | |
|  |  | Size-Zone Non-Uniformity (SZN) | |
|  |  | Size-Zone Non-Uniformity Normalized (SZNN) | |
|  |  | Small Area Emphasis (SAE) | |
|  |  | Small Area High Gray Level Emphasis (SAHGLE) | |
|  |  | Small Area Low Gray Level Emphasis (SALGLE) | |
|  |  | Zone Entropy (ZE) | |
|  |  | Zone Percentage (ZP) | |
|  |  | Zone Variance (ZV) | |
| **Textural Features: Gray Level Dependence Matrix (****GLDM) Features** |  | Dependence Entropy (DE) | |
| **(N=14)** |  | Dependence Non-Uniformity (DN) | |
|  |  | Dependence Non-Uniformity Normalized (DNN) | |
|  |  | Dependence Variance (DV) | |
|  |  | Gray Level Non-Uniformity (GLN) | |
|  |  | Gray Level Variance (GLV) | |
|  |  | High Gray Level Emphasis (HGLE) | |
|  |  | Large Dependence Emphasis (LDE) | |
|  |  | Large Dependence High Gray Level Emphasis (LDHGLE) | |
|  |  | Large Dependence Low Gray Level Emphasis (LDLGLE) | |
|  |  | Low Gray Level Emphasis (LGLE) | |
|  |  | Small Dependence Emphasis (SDE) | |
|  |  | Small Dependence High Gray Level Emphasis(SDHGLE) | |
|  |  | Small Dependence Low Gray Level Emphasis (SDLGLE) | |

**S3:** **Methods of each step in the radiomics pipeline.**

The model exploration pipelines were accomplished using an open-source software package (FAE) (3) developed in Python v 3.6 ([https://python.org](https://python.org/)) and a Qt framework. Most algorithms in FAE were implemented with scikit-learn 0.19 ([https://scikit-learn.org](https://scikit-learn.org/)). The source code is openly available on GitHub (<https://github.com/salan668/FAE.git>).

**Table S3 Available options for each step in the radiomics model development pipeline**

| Steps |  | Methods |
| --- | --- | --- |
| Normalization |  | None |
|  |  | Min-Max |
|  |  | Z-score |
|  |  | Mean |
| Dimension Reduction |  | Principle Component Analysis (PCA) |
|  |  | Pearson Correlation Coefficient (PCC) |
| Feature Selection |  | Analysis of Variance (ANOVA) |
|  |  | Recursive Feature Elimination (RFE) |
|  |  | Relief |
|  |  | Kruskal-Wallis Test |
| Classification |  | Support Vector Machine (SVM) |
|  |  | Linear Regression (LR) |
|  |  | Least Absolute Shrinkage and Selection Operator (LASSO) |
|  |  | Linear Discriminant Analysis (LDA) |
|  |  | Decision Tree (DT) |
|  |  | Random Forest (RF)  eXtreme Gradient Boosting (XGB) |
|  |  | Adaboost |
|  |  | Gaussian Process |
|  |  | Naïve Bayes |
|  |  | Multilayer Perceptron |

**S4:** **Details of the radiomics modeling pipelines**

*Normalization: Mean*

Another method of mean normalization feature scaling. The function of feature scaling and mean normalization is to reduce the fluctuation of sample data so that gradient descent can find a "shortcut" more quickly, to reach the global minimum. Therefore, mean normalization is to get the mean of all samples first, so that the change of sample data is equally obvious in a smaller range.

*Normalization: Z-score*

The Z-score technique was used to obtain a standard normal distribution in the datasets. The mean value and the standard deviation were calculated. Each feature vector was subtracted from the mean value and divided by the standard deviation. After the Z-score process, each vector had a zero center and unit standard deviation.

*Dimension reduction: PCA*

Since the dimension of the feature space was high, a principal component analysis (PCA) of the feature matrix was performed to reduce the data dimensionality. During this process, by linear combinations, the original features were reidentified as new variables, i.e., principal components (4). Then, the feature vector of the transformed feature matrix was independent of each other.

*Dimension reduction: PCC*

To reduce the dimensions of the row space of the feature matrix, a Pearson correlation coefficient (PCC) was applied to each pair of two features. We compared the similarity of each feature pair. If the PCC value of the feature pair was larger than 0.99, we removed one feature randomly. After this process, the dimension of the feature space was reduced, and each feature was independent of the other features.

*Feature selection: ANOVA*

Analysis of variance (ANOVA) was used to select the features before building the model. ANOVA is a common method used to explore the significant features corresponding to the labels. The F-value was calculated to evaluate the relationship between the features and the label. We sorted the features according to the corresponding F-value and selected a specific number of features to build the model.

*Feature selection: KW*

The Kruskal-Wallis test was used to select the features before building the model. The Kruskal-Wallis test is a common method used to explore the significant features corresponding to the labels, and all features had corresponding *P*-values. If the features had *P*-values smaller than 5%, the features were significant for the corresponding label, had value for further analysis, and were reserved.

*Classifier: XGB*

*XGBoost is an optimized distributed gradient boosting library designed to be highly efficient, flexible, and portable. It implements machine learning algorithms under the Gradient Boosting framework. XGBoost provides a parallel tree boosting (also known as GBDT, GBM) that solves many data science problems in a fast and accurate way. The same code runs on a major distributed environment (Hadoop, SGE, MPI) and can solve problems beyond billions of examples.*

*Classifier: DT*

Decision Trees (DTs) are a non-parametric supervised learning method used for classification and regression. The goal is to create a model that predicts the value of a target variable by learning simple decision rules inferred from the data features. A tree can be seen as a piecewise constant approximation. Decision trees learn from data to approximate a sine curve with a set of if-then-else decision rules. The deeper the tree, the more complex the decision rules, and the fitter the model.

*Classifier: RF*

Random forest is a commonly-used machine learning algorithm trademarked by Leo Breiman and Adele Cutler, which combines the output of multiple decision trees to reach a single result. Its ease of use and flexibility have fueled its adoption, as it handles both classification and regression problems. Random forest algorithms have three main hyperparameters, which need to be set before training. These include node size, the number of trees, and the number of features sampled. From there, the random forest classifier can be used to solve regression or classification problems.

**S5: Equations for radiologiacal model**

$p={e^{x}}/\left( 1+e^{x} \right)$, $x=\left( 1.736\times irregular margin of tumor thrombus \right)+\left( 3.949\times abnormal signal intensity on T 2WI \right)-1.812$

According to assessment of MR images: irregular margin of tumor thrombus, abnormal signal intensity on T 2WI (0: absent, 1: present). There should be only 4 patterns of probabilities, namely, presence or absence of margin irregularity (condition A), and abnormal signal within IVC wall (condition B) (2x2=4), which would be 0.140397, 0.979628, 0.894448, and 0.481009, for A-&B-, A+&B+, A-&B+, and A+&B-, respectively.

**S6:** **Key features and their F value in each model**

| Model | Key features | F | p |
| --- | --- | --- | --- |
| model 1 | PCA_feature_1 | 9.742 | 0.002 |
|  | PCA_feature_3 | 4.127 | 0.042 |
|  | PCA_feature_4 | 5.292 | 0.021 |
|  | PCA_feature_6 | 4.988 | 0.026 |
|  | PCA_feature_8 | 5.543 | 0.019 |
| model 2 | original_shape_MinorAxisLength | 20.455 | ＜0.001 |
|  | original_shape_Maximum2DDiameterSlice | 21.535 | ＜0.001 |
|  | wavelet-LLL_glszm_GrayLevelNonUniformity | 18.773 | ＜0.001 |
|  | original_glszm_GrayLevelNonUniformity | 18.802 | ＜0.001 |
| model 3 | PCA_feature_1 | 9.742 | 0.002 |
|  | PCA_feature_3 | 4.127 | 0.042 |
|  | PCA_feature_4 | 2.292 | 0.021 |
|  | PCA_feature_6 | 4.988 | 0.026 |
|  | PCA_feature_8 | 5.543 | 0.019 |
|  | PCA_feature_16 | 2.634 | 0.100 |
|  | PCA_feature_33 | 3.491 | 0.061 |
|  | PCA_feature_44 | 3.397 | 0.065 |
|  | PCA_feature_62 | 3.106 | 0.078 |
| model 4 | original_shape_MinorAxisLength | 20.455 | ＜0.001 |
|  | original_shape_Maximum2DDiameterSlice | 21.535 | ＜0.001 |
|  | wavelet-LLL_glszm_GrayLevelNonUniformity | 18.773 | ＜0.001 |
|  | wavelet-LLL_glrlm_GrayLevelNonUniformity | 18.490 | ＜0.001 |
|  | log-sigma-5-0-mm-3D_gldm_GrayLevelNonUniformity | 18.186 | ＜0.001 |
|  | original_glcm_Imc2 | 17.964 | ＜0.001 |
|  | original_glszm_GrayLevelNonUniformity | 18.802 | ＜0.001 |
|  | Maximal_coronal_diameterofIVC | 25.868 | ＜0.001 |
|  | Irregular_marginoftumor_thrombus_0 | 34.092 | ＜0.001 |
|  | Abdomal_signal_intensity_on_T2WI_0 | 84.751 | ＜0.001 |

model 1 = radiomics model_IVC; model 2= radiomics model_TT; model 3 = combined model_IVC; model 4 = combined model_TT; model 5 = radiological model

**Reference**

1. community P. Customizing the Extraction (2016) [cited 2021 Jan 10]. Available from: <https://pyradiomics.readthedocs.io/en/latest/customization.html>.

2. community P. Radiomics Feature Extraction in Python (2016) [cited 2020 Oct 23]. Available from: <https://github.com/radiomics/pyradiomics>.

3. Yang Song JZ, Yu-Dong Zhang, Ying Hou, Xu Yan, Yida Wang, Minxiong Zhou, Ye-Feng Yao, Guang Yang. Feature Explorer (Fae): A Tool for Developing and Comparing Radiomics Models. *PLoS One* (2020) 15(8):e0237587. Epub 2020/08/18. doi: 10.1371/journal.pone.0237587.

4. Fangying Chen XM, Shuai Li, Zhihui Li, Yan Jia, Yuwei Xia, Minjie Wang, Fu Shen, Jianping Lu. Mri-Based Radiomics of Rectal Cancer: Assessment of the Local Recurrence at the Site of Anastomosis. *Acad Radiol* (2020) S1076-6332(20):30567-5. doi: 10.1016/j.acra.2020.09.024.
